# Psychological aspects of nurses’ attitudes towards death

**DOI:** 10.1192/j.eurpsy.2025.1814

**Published:** 2025-08-26

**Authors:** A. M. Cybulska, K. Rachubińska, D. Schneider-Matyka, I. Czeryna, E. Grochans

**Affiliations:** 1 Department of Nursing, Pomeranian Medical University in Szczecin; 2 Independent Public Provincial Hospital in Szczecin, Szczecin, Poland

## Abstract

**Introduction:**

Attitude towards death is an individual’s psychological reaction to death, which includes basic components, such as thinking about death, fear of death, and defence mechanisms. Due to the nature of their work, nurses often accompany patients at the end of life and choose different ways to cope with this difficult situation.

**Objectives:**

The aim of this study was to analyze the psychological aspects of nurses’ attitudes towards the death of a patient, and to determine the variables that influence their choice of strategies to deal with this difficult situation.

**Methods:**

This survey-based study was performed using the author questionnare, and standardized research tools, namely *the Polish adaptation of* the Death Attitude Profile Revised (DAP-R-PL), and the Mini-COPE inventory.

**Results:**

The study involved 315 subjects with a license to practice nursing, 85.7% of whom were women. The age of the respondents ranged from 21 to 70 years, and was on average 40.5 years (SD = 10.7). The nurses’ dominant attitudes towards death were neutral acceptance (5.40 ± 0.97 points) and escape acceptance (5.09±1.01 points). Age, sex, work experience, and having a specialty credential influenced the attitudes towards death according to the DAP-R-Pl. The predominant stress coping strategies among the nurses according to the Mini-COPE were: self-distraction (1.98 ± 0.75 points), active coping (1.88 ± 0.69 points), planning (1.87 ± 0.77 points), and acceptance (1.84 ± 0.62 points). Factors that had an impact on the choice of stress coping strategies according to the Mini-COPE were: sex, place of residence, marital status, education, having a specialty credential, work experience, and place of work. Based on the collected data, statistically significant correlations were also found between the nurses’ attitudes towards death according to the DAP-R-PL and stress coping strategies according to the Mini-COPE.

**Conclusions:**

The nurses showed different attitudes towards the death of a patient, the most common of which were neutral acceptance and escape acceptance. Age, sex, marital status, and place of residence had a significant impact on the nurses’s attitudes towards the patient’s death. Contact with dying patients evokes many negative emotions in nurses, such as sadness, compassion, helplessness, regret, and a sense of injustice. Therefore, it is important to provide them with psychological support when not coping with the death of a patient. Due to the strong emotional reaction associated with the death of a patient, nurses choose coping strategies, such as: self-distraction, active coping, planning, and acceptance. Both sociodemographic and work-related variables influence the choice of coping strategies for the patient’s death.

**Disclosure of Interest:**

None Declared

